# Platelet/High-Density Lipoprotein Ratio (PHR) Predicts Type 2 Diabetes in Obese Patients: A Retrospective Study

**DOI:** 10.3390/healthcare12151540

**Published:** 2024-08-03

**Authors:** Yazeed Alshuweishi, Arwa Abudawood, Dalal Alfayez, Abdulmalik A. Almufarrih, Fuad Alanazi, Fahd A. Alshuweishi, Abdulaziz M. Almuqrin

**Affiliations:** 1 Department of Clinical Laboratory Sciences, College of Applied Medical Sciences, King Saud University, Riyadh 12372, Saudi Arabia; foalanazi@ksu.edu.sa (F.A.); aalmuqrin@ksu.edu.sa (A.M.A.); 2 Department of Family and Community Medicine, Prince Sultan Military Medical City, Riyadh 11159, Saudi Arabia; aabudawood@psmmc.med.sa (A.A.); dalfayez@psmmc.med.sa (D.A.); aalmufarrih@psmmc.med.sa (A.A.A.); 3 King Fahad Kidney Center, King Saud Medical City, Riyadh 12746, Saudi Arabia; falshuweishi@ksmc.med.sa

**Keywords:** obesity, T2D, platelets, HDL, hyperglycemia, inflammation

## Abstract

**Background:** Obesity and type 2 diabetes (T2D) pose global health problems that continue to rise. A chronic low-grade inflammation and activation of the immune system are well established in both conditions. The presence of these factors can predict disease development and progression. Emerging evidence suggests that platelet–high density lipoprotein ratio (PHR) is a potential inflammatory marker. The purpose of this study was to investigate the relationship between PHR and T2D among obese patients. **Methods:** 203 patients with BMI ≥ 30 kg/m^2^ participated in the study. Patients were categorized into two groups: non-diabetic obese and diabetic obese. Comorbidities, baseline characteristics, laboratory data, as well as PHR levels of the study groups were analyzed. Medians, risk assessment, and the diagnostic performance of PHR values were examined in both groups. **Results:** In obese patients, the median PHR were significantly increased in obese patients with T2D compared to non-diabetic obese (*p* < 0.0001). Furthermore, T2D obese with high PHR had a significantly higher FBG and HbA1c (*p* < 0.05). Although PHR was weakly yet significantly correlated with glycemic markers, ROC curve analysis of the PHR indicated an AUC of 0.700 (*p* < 0.0001) in predicting T2D in obese patients, and the cutoff value was 6.96, with a sensitivity and specificity of 53.4% and 76.1%, respectively. Moreover, increased PHR (OR = 4.77, *p* < 0.0001) carried a significantly higher risk for developing T2D in obese individuals. **Conclusions:** The PHR is a convenient and cost-effective marker that can reliably predict the presence of T2D in high-risk obese population.

## 1. Introduction

Obesity is an urgent global health issue, with Saudi Arabia showing alarmingly high rates. Recent data indicate that over 50% of the Saudi population is overweight, and more than 20% are obese [1,2]. This trend is largely driven by rapid urbanization and economic growth, leading to less physical activity and higher consumption of high-calorie diets [3]. The high prevalence of obesity not only poses a significant burden on the healthcare system but also impacts the overall quality of an individual’s life [3,4].

One of the significant concerns linked to obesity is the increased prevalence of type 2 diabetes (T2D), a condition closely associated with excessive body weight [5]. Obesity is known as a triggering factor for the development of T2D mainly due to the increased insulin resistance that accompanies excess body fat [6]. When BMI increases from 18.5 kg/m^2^ to over 35 kg/m^2^, the risk of developing T2D over the age of 18 years rises dramatically from 7% to 70% in males and from 12% to 74% in females [7]. The cellular and physiological mechanisms responsible for the link between obesity and T2D are complex and involve β cell dysfunction, alteration in adipose tissue biology, and multi-organ insulin resistance [8,9]. Although a vast majority of T2D patients are overweight or obese, not all obese individuals develop T2D, suggesting that multiple factors are involved in the development of T2D [7,10]. This includes genetics, lifestyle, and various inflammatory responses, with an increasing body of evidence supporting a key role of inflammation in obesity as well as T2D [11]. However, it is still challenging to distinguish specific inflammatory markers associated with obesity or T2D. Studies to identify distinctive inflammatory biomarkers that uniquely characterize each of the two conditions and identify diabetes risk in obese individuals are crucial for timely intervention and prevention of disease progression. Therefore, early screening for diabetes in all obese individuals is highly recommended.

During inflammatory responses, the number of neutrophils, monocytes, lymphocytes and platelets changes [12,13,14,15]. Among these, platelets, traditionally known for their role in homeostasis, play a critical role in inflammation by interacting with leukocytes and endothelial cells, and by releasing various inflammatory mediators [16,17,18]. It was shown that elevated platelet counts were associated with inflammation severity and treatment outcomes in patients with ankylosing spondylitis, inflammatory bowel disease, Crohn’s disease, rheumatoid arthritis and systemic lupus erythematosus [19,20,21,22,23]. In the context of T2D, increased platelet reactivity was observed in T2D patients with significant association between HbA1c levels and platelet number [24,25,26]. On the other hand, high-density lipoprotein (HDL) is well known for its role in reverse cholesterol transport, but it also exhibits significant anti-inflammatory properties [27]. These anti-inflammatory actions include removing cholesterol from macrophages in atherosclerotic plaques, reducing the formation of foam cells and subsequent inflammatory responses [28]. Additionally, HDL inhibits the oxidation of low-density lipoprotein (LDL), thereby preventing the pro-inflammatory signaling triggered by oxidized LDL [29]. Consequently, reduced HDL levels were substantially associated with an increased risk of major adverse cardiovascular events in T2D obese patients [30,31].

Therefore, recent studies have utilized both platelet counts and HDL as a critical indicator of inflammatory responses in various diseases [32,33]. Jialal et al. revealed that the platelet to HDL-C ratio (PHR) is significantly increased in patients with metabolic syndrome and appears to be a valid biomarker of metabolic syndrome [34]. As metabolic syndrome is generally associated with T2D, it is possible that the PHR may be able to be used as a biomarker in predicting T2D in at-risk populations such as obese subjects. Nevertheless, the relationship between PHR level and obese patients with or without T2D remains unexplored. Therefore, this study was designed to understand the relationships between the blood-derived inflammatory marker PHR and the occurrence of T2D in obese patients. The current research also aimed to investigate the association estimate and the diagnostic accuracy of PHR for markers of glucose metabolism among diabetic and non-diabetic obese patients.

## 2. Methods

### 2.1. Study Design and Data Collection

In this study, data from a total of 203 patients were collected between 2022 and 2023 from Family Medicine Clinics at the Prince Sultan Military Medical City (PSMMC), Riyadh, Saudi Arabia. This included 103 non-diabetic obese patients from the Family Medicine Lifestyle Clinic and 100 diabetic obese patients from the Family Medicine Chronic Illnesses Clinic. Approval for this study was obtained from the Institutional Review Board (IRB) at PSMMC (IRB number: E-2165, approved on 14 September 2023). Type 2 diabetes (T2D) was confirmed by a physician and data were available from patient hospital records. According to hospital protocol, T2D was diagnosed either by a FBG level ≥ 126 mg/dL, a 2 h plasma glucose tolerance test of ≥200 mg/dL, or a HbA1c ≥ 6.5%, confirmed by a repeated test on a different day [35]. Obesity was defined as a body mass index (BMI) ≥ 30 kg/m^2^ [36]. Patients were excluded if they were younger than 18 years of age, had type 1 diabetes, or were pregnant.

For further analysis, the subjects from each group were classified according to their obesity into three classes:

- Obesity Class I (BMI 30 to 34.9 kg/m^2^)
- Obesity Class II (BMI 35 to 39.9 kg/m^2^)
- Obesity Class III (BMI ≥ 40 kg/m^2^)

PHR was calculated as platelet count (×10^6^/mL) ÷ HDL cholesterol (mg/dL) [34]. The optimal cut-off value for PHR value was >6.96 based on the best cutoff (i.e., highest sensitivity and specificity) as revealed by receiver operating characteristics (ROC) curve analysis. Therefore, PHR values > 6.96 were considered high. Laboratory data, including PHR levels, were then analyzed to assess the patterns of PHR among non-diabetic and diabetic obese patients.

### 2.2. Statistical Analysis

Since the collected data were not normally distributed, nonparametric tests were used for statistical analysis. One-way ANOVA with the Kruskal–Wallis test was used to compare between three study groups while the Mann–Whitney U test was used to compare two study groups. The analyzed data were displayed as medians ± interquartile range (IQR). The predictive power of PHR to discriminate T2D was assessed by ROC curve analysis and area under the curve (AUC). Simple linear regression analysis was utilized to assess the association between PHR and other variables. The statistical analysis was performed using GraphPad Prism v9.2.0 (GraphPad Software, Inc., 10.0.01, San Diego, CA, USA). *p*-values below 0.05 were considered statistically significant.

## 3. Results

### 3.1. Baseline Characteristics of the Studied Population

This retrospective study included 203 obese participants, 141 of whom were females. The studied population was stratified into two groups: obese with T2D (diabetic obese) group and obese without T2D group (non-diabetic obese). The average age of the diabetic obese group was 57.5 years (±51.25–64.75), whereas the average age of the non-diabetic obese group was 37 years (±29–46). The laboratory parameters showed significant differences between the diabetic obese and the non-diabetic obese groups for some parameters as illustrated in Table 1. WBC counts, neutrophils, lymphocytes, monocytes and basophils were all significantly higher in the diabetic obese group. Almost 30% of the studied subjects were hypertensive, and most of them belonged to the diabetic obese group (Table 1). The number of smokers in the studied population was 17 (16.5%) and 5 (5%) for the non-diabetic obese and diabetic obese, respectively. 77% of diabetic obese patients were dyslipidemic, whereas 18.4% of non-diabetic obese patients had dyslipidemia. As demonstrated in Table 1, Metformin was used by 93% of individuals in the diabetic obese group, while only 10.6% of subjects in the non-diabetic obese group took this medicine. In addition, 26% and 34% of the diabetic obese patients used GLP-1 agonist and SGL2 inhibitor, respectively. On the other hand, 30% of non-diabetic obese subjects used GLP-1 and no participant from the same group used SGL2 inhibitor.

### 3.2. PHR Is Substantially Increased in Obese T2D Patients with a Significant Diagnostic Performance in Predicting T2D in the Studied Population

**Figure 1:**
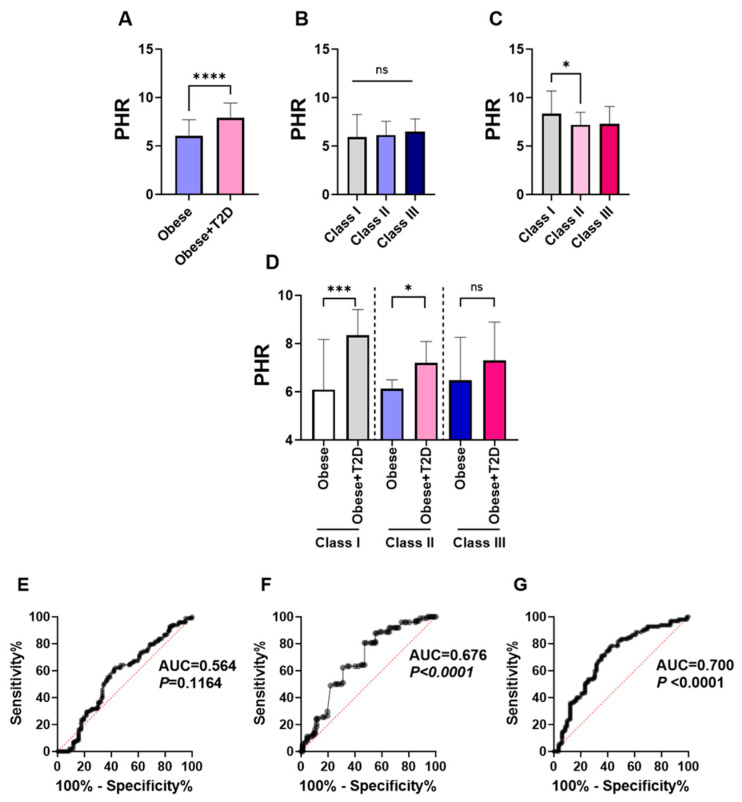
Comparison of PHR levels between non-diabetic and diabetic obese groups and ROC curve of PHR for T2D prediction. Changes in PHR levels between non-diabetic and diabetic obese groups are presented in (**A**). PHR values according to obesity classes for non-diabetic obese (**B**) and diabetic obese (**C**) and both groups (**D**) are shown. ROC curve analysis of PLT count (**E**), HDL levels (**F**), and PHR (**G**) to distinguish T2D among obese patients are illustrated. ns indicates not significant while * (*p* < 0.05), *** (*p* < 0.001), and **** (*p* < 0.0001).

The changes in the levels of PHR index in the studied groups were analyzed as illustrated in (Figure 1A). The results showed a significant increase in PHR levels in the diabetic obese (7.910 ± 6.390–9.435) compared to the non-diabetic obese group (6.050 ± 4.745–7.718). Then we evaluated the possible association between the severity of obesity and the levels of PHR within each studied group.

The obese subjects were stratified into three groups according to obesity severity: Obesity class I, Obesity class II, and Obesity class III (Supplemental Table S1). Although statistical significance was not achieved, our findings exhibited a proportional increase in PHR according to the severity of obesity in the non-diabetic obese group. (PHR; 5.93 ± 4.645–8.24 vs. 6.13 ± 4.72–7.54 vs. 6.475 ± 5.59–7.81) (Figure 1B). Interestingly, when the same analysis was performed in the diabetic obese group, we observed a significant increase in PHR levels in obesity class I subjects (8.340 ± 6.990–10.68) compared to obesity class II subjects (7.200 ± 5.710–8.485) (Figure 1C). In addition, the analysis revealed an increase in PHR levels among obesity class I subjects compared to those classified within obesity class III; however, statistical significance was not obtained (Figure 1C).

Nevertheless, when comparing PHR values between non-diabetic obese and diabetic obese patients within each obesity class (Figure 1D), PHR levels were significantly higher in diabetic obese class I and II compared to their non-diabetic obese counterparts (Class I, 8.34 ± 6.99–10.68 vs. 6.09 ± 4.69–8.48; Class II, 7.20 ± 5.71–8.49 vs. 6.13 ± 4.72–7.54). Obesity class III, despite a trend toward higher PHR levels in diabetic obese class III, did not achieve statistical significance (Figure 1D; 7.295 ± 5.918–9.085 vs. 6.48 ± 5.59–7.81). Although the reasons for the lack of significant difference in the class III group are not known, they might be related to the small size number, especially in non-diabetic obese III patients (Supplemental Table S1), who made up 12.6% of all non-diabetic obese patients.

The receiver operating characteristic (ROC) curve of PLT was performed to evaluate its predictive ability in discriminating T2D subjects in the studied population. The results demonstrated a poor predictive ability of the PLT count for T2D in the studied population (AUC = 0.564, *p* = 0.116) (Figure 1E). Following that, the diagnostic ability of the HDL marker for T2D was assessed. HDL demonstrated a markedly better predictive ability for T2D compared to the platelet count (AUC = 0.67, *p* < 0.0001) (Figure 1F). Finally, the combined predictive capability of the platelet to HDL ratio (PHR) for T2D was evaluated. This combined marker demonstrated superior performance in the ROC curve analysis for T2D prediction compared to the PLT count and HDL markers (AUC = 0.700, *p* < 0.0001) (Figure 1G).

### 3.3. FBG and HbA1c Were Significantly Higher in High-PHR Obese Patients with T2D

**Figure 2:**
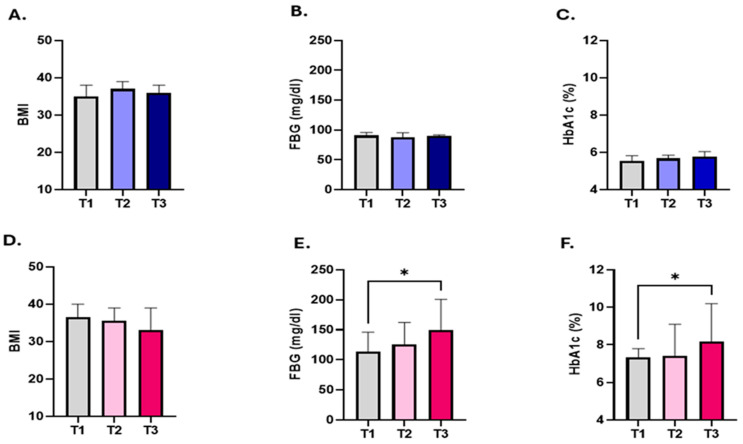
Evaluating the BMI, FBG, and HbA1c levels in relation to PHR in the studied population. The non-diabetic and diabetic obese groups were categorized based on their PHR levels into three distinct tertiles (T1, T2, and T3). Medians ± IQR of BMI (**A**), FBG (**B**), and HbA1c (**C**) in relation with each tertile in the non-diabetic are presented. Medians ± IQR of BMI (**D**), FBG (**E**), and HbA1c (**F**) according to each tertile in the in diabetic obese group are shown. * indicates (*p* < 0.05).

To analyse the association between BMI, FBG, and HbA1c with PHR levels in the studied population, patients within both the diabetic obese and non-diabetic obese groups were classified based on their PHR levels into three distinct tertiles (T1, T2, and T3). T1 includes subjects with the lowest values of PHR, representing the metric’s minimum range. T2 encompasses the PHR values of subjects that fall within the intermediate range, while T3 contains subjects with the highest PHR values. As shown in Figure 2A–C, no significant difference was observed in patterns of BMI, FBG, and HBA1c between the T1, T2, and T3 tertiles within the non-diabetic obese group. In contrast, we observed a significant difference between T1 and T3 in light of FBG (113.5 ± 93.69–145.9 vs. 149.5 ± 113.5–200.9) and HbA1c (7.320 ± 6.425–7.800 vs. 8.170 ± 7.200–10.20) within the diabetic obese group, while no statistically significant was observed between T1, T2, and T3 in the context of BMI (Figure 2D–F).

### 3.4. Correlation of PHR Values with BMI and Glycemic Parameters, FBG and HbA1c

**Figure 3:**
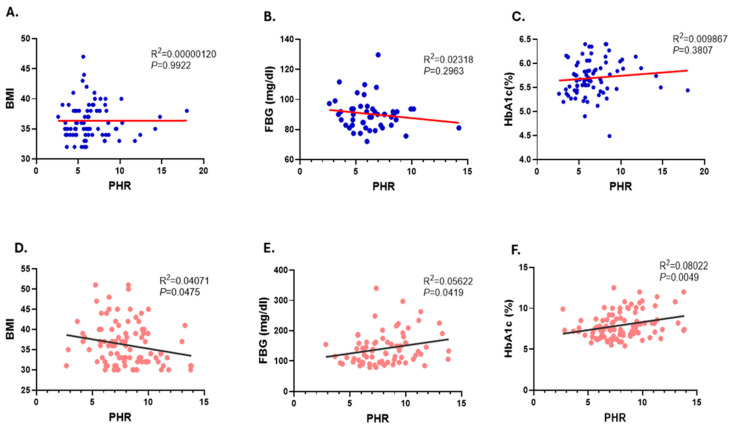
Association of PHR with BMI, FBG, and HbA1c in the studied subjects. Simple linear regression of the association of PHR with BMI (**A**), FBG (**B**), and HbA1c (**C**) in non-diabetic subjects. Simple linear regression of the association of PHR and BMI (**D**), FBG (**E**), and HbA1c (**F**) in diabetic obese subjects.

Furthermore, our linear regression analysis found no significant correlation between the PHR levels and BMI, FBG, and HbA1c in the non-diabetic obese group (Figure 3A–C). In contrast, our observation revealed a significant correlation between the PHR levels and BMI (R^2^ = 0.0407 and *p* = 0.0475), FBG (R^2^ = 0.05622 and *p* = 0.0419), and HbA1c (R^2^ = 0.08022 and *p* = 0.0049) in diabetic obese subjects, as shown in (Figure 3D–F).

**Figure 4:**
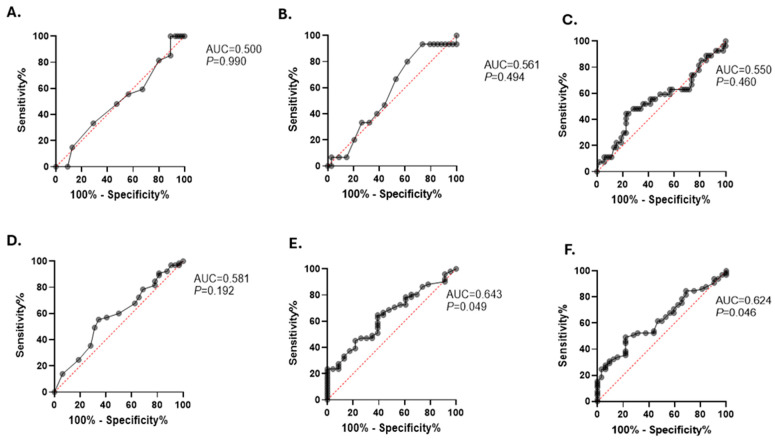
Evaluating the ability of elevated PHR to predict BMI, FBG, and HbA1c markers in the studied population. ROC curves of the elevated PHR and BMI (**A**), FBG concentrations (**B**), and HbA1c levels (**C**) for the non-diabetic obese participants are shown. ROC curves of the elevated PHR and BMI (**D**), FBG concentrations (**E**), and HbA1c levels (**F**) for the diabetic obese subjects are illustrated.

Then, we compared the capability of elevated PHR to predict BMI, FBG, and HbA1c. In the non-diabetic obese group, PHR did not significantly differentiate patients with elevated FBG or HbA1c levels (Figure 4B,C). In contrast, in the diabetic obese group, PHR demonstrated a stronger capability to discriminate between patients with increased FBG (AUC = 0.643, *p* = 0.049) and higher HbA1c levels (AUC = 0.624, *p* = 0.046), as shown in Figure 4E,F. We acknowledge that the statistical significance (*p* = 0.049) is marginal; however, our findings indicate that PHR’s predictive capacity for dysglycemia is notably better in the context of diabetic obesity compared to non-diabetic obesity.

### 3.5. Elevated PHR Is Associated with an Increased Risk of T2D in Obese Patients

As demonstrated in Table 2, the prevalence of elevated PHR levels was considerably higher among the diabetic obese (70.10%) compared to the non-diabetic obese patients (32.93%). In addition, our analysis revealed that 67.07% of non-diabetic obese had normal levels of PHR. This percentage reduced to 29.90% within the diabetic obese group.

The risk assessment analysis revealed an association between the elevated levels of PHR and occurrence of T2D in the overall studied population (Table 3, RR = 2.13, 95% CI: 1.52 to 2.97, *p* < 0.0001). Similar results were obtained for subjects that were classified under obesity class I (RR = 2.12, 95% CI: 1.23 to 3.67, *p* = 0.0067). The analysis showed the risk of increased PHR in developing T2D within obese class II participants was 1.60 (RR = 1.6, 95% CI: 0.86 to 2.97, *p* = 0.1345). Nonetheless, statistical significance was not obtained. On the other hand, our observation suggested that the elevation in PHR levels for those classified under obesity class III was associated with a 42% reduction in the risk of developing T2D (RR = 0.58, 95% CI: 0.41 to 0.81, *p* = 0.0018). Additionally, the results showed that increased PHR levels in the overall studied population, obesity class I subjects, and obesity class II participants were 4.77, 5.81, and 5.62 times more likely to fall into the T2D group, respectively.

## 4. Discussion

A quarter of Saudi Arabia’s population suffers from diabetes mellitus (DM), a severe and common inflammatory disease that is predicted to quadruple in prevalence by 2030 [37]. Prognostic and monitoring biomarkers are highly desired in the clinical setting to predict individuals at high risk for developing T2D. The current study was undertaken to demonstrate the link between blood-derived PHR and the occurrence of T2D among obese individuals. For obese patients, including non-diabetic and diabetic obese patients, we showed for the first time that PHR was significantly elevated among diabetic obese participants compared to the non-diabetic obese group. Additionally, ROC curve analysis revealed that PHR was able to differentiate between non-diabetic obese and diabetic obese. Particularly among diabetic obese patients, FBG and HbA1c were significantly higher in the highest PHR tertile, compared with patients in the other tertiles. In line with this, PHR was significantly associated with glycemic parameters FBG and HbA1c in diabetic but not non-diabetic obese patients. These findings demonstrate differential patterns of PHR among non-diabetic and diabetic obese patients, suggesting that PHR could serve as a marker of dysglycemia.

There is a paucity of studies involving PHR and its relationship with obesity and T2D. Comparing obese patients with and without T2D showed that PHR levels were significantly elevated, with an approximately 25% increase among diabetic groups. The ability of the PHR to discriminate diabetic obese from non-diabetic obese suggests that the PHR may be a sensitive marker for predicting T2D among high-risk populations such as obese patients. However, our findings suggest that the severity of obesity does not seem to be associated with PHR, since PHR levels were not significantly altered when obese patients were stratified into class I, II, or III obesity, either in obese or diabetic obese groups. Nevertheless, when comparing PHR values between non-diabetic and diabetic obese patients within each obesity class, we observed significantly higher PHR levels in diabetic obese class I and II compared to their non-diabetic counterparts. However, this difference was not observed in class III, where we noted a trend toward higher PHR levels that did not achieve statistical significance. Moreover, the risk assessment analysis revealed that elevated levels of PHR had higher odds of T2D in the overall studied population as well as in obesity class I and II but not class III. This lack of significant effects of elevated PHR in class III may be related to a small sample size, particularly among non-diabetic obese class III patients, who constituted only 12.6% of all non-diabetic obese patients. Additionally, there are conflicting data regarding the relationship between platelet counts and body mass index (BMI), which might contribute to the observed variations in PHR levels among different obesity classes. A number of studies showed that platelet counts were altered in subjects with high BMI [38,39,40], while Coban et al. reported normal platelet counts in the obese individuals when compared to non-obese normal individuals [41]. Based on these findings, it appears that the observed elevation of PHR levels in T2D might not be related to obesity status; however, further longitudinal studies are certainly warranted.

One important finding in this study is the significant association of PHR with glycemic parameters among T2D patients. The potential reasons for such an association might be related to the role of individual components of PHR, platelets and HDL in light of T2D. Indeed, it was reported that patients with T2D exhibited higher platelet counts compared to non-diabetic controls [42]. Moreover, an early study by Gordon et al. followed by more recent studies have shown that diabetic patients typically exhibit lower HDL levels compared to non-diabetic controls [43,44,45]. This relationship might be due to the chronic inflammatory state induced by hyperglycemia, the hallmark metabolic abnormality associated with T2D. Indeed, we observed a significant increase in numbers of total leukocytes, neutrophils, lymphocytes, monocytes, and basophils in the diabetic obese group compared to the non-diabetic obese group, indicating ongoing chronic inflammation associated with T2D. Moreover, the elevated leukocyte counts observed in our study suggest a persistent immune activation, which is known to exacerbate insulin resistance and further impair glucose metabolism. The specific increase in neutrophils and monocytes may reflect an enhanced innate immune response, which has been linked to endothelial dysfunction and the development of cardiovascular diseases commonly seen in T2D patients. Elevated lymphocytes, on the other hand, indicate adaptive immune system involvement, which has been implicated in the autoimmune aspects of diabetes pathogenesis [46]. Notably, chronic hyperglycemia induces a proinflammatory state that has detrimental effects on the hematopoietic system, leading to increased megakaryocyte proliferation and platelet production [47]. The persistent high glucose levels enhance platelet turnover and activation, contributing to the observed hematological changes [48]. Moreover, Kodiatte et al. demonstrated that HbA1c levels were positively correlated with the mean platelet volume (MPV), a marker of platelet activation, suggesting that poor glycemic control leads to greater platelet activation [49]. Insulin resistance, a hallmark of T2D, also contributes to changes observed in platelet number and function [50]. Under normal conditions, insulin inhibits platelet aggregation by suppressing the P2Y_12_ pathway in healthy volunteers [51]; however, such antithrombotic protection is hampered in T2DM patients due to reduced insulin sensitivity by platelets [52]. Furthermore, reactive oxygen species (ROS), commonly observed in diabetes, also play a critical role in modifying platelet function and increasing platelet counts in diabetic conditions [53,54]. Based on the above reports regarding the role of platelets on glycemic parameters, we could infer that platelet count or function might be unfavorable prognostic factors in the context of dysglycemia and diabetes.

It is well documented that individuals with T2D often have reduced levels of HDL cholesterol largely due to the altered metabolism of lipids seen in T2D conditions [55]. It was shown that insulin normally promotes the production of apolipoprotein A-I (ApoA-1), a major component of HDL [56]. This event is impaired in insulin-resistant states leading to lower HDL levels [57,58]. Additionally, the glycation process has been proposed as one of the mechanisms explaining the link between HDLs, inflammation, and diabetes. It was demonstrated that hyperglycemia causes glycation of the ApoA-1 molecule of HDL particles, leading to an increase in its degradation three times faster than in a person without diabetes [59]. Beyond the reduction in quantity, HDL functionality is also compromised in diabetes, due to oxidative modification and glycation of the HDL protein. It has been reported that glycated HDL was not able to reduce in vitro palmitate-induced inflammation in cultured adipocytes, likely due to increased serum amyloid A (SAA) concentrations in HDL following inflammation [60]. In diabetic nephropathy, increased SAA levels were directly associated with impaired HDL capacity to reduce the secretion of TNF-α from human peripheral blood mononuclear cells (PBMCs) [61]. Mechanistically, SAA enrichment causes displacement of paraoxonase 1 (PON1) and ApoA-I from HDL particles, potentially leading to HDL dysfunction [62]. A recent proteomics study comparing serum HDL from control and diabetic individuals revealed that the diabetic HDL had significantly higher glycated ApoA-I [59], suggesting that this parameter could be considered a valuable diagnostic tool to assess the metabolic state of diabetic patients. These findings clearly suggest how inflammation along with hyperglycemia might contribute to HDL perturbation in the light of T2D. Therefore, our findings showing elevated levels of PHR in diabetic patients might be a result of reduced HDL levels.

The incidence of T2D is rapidly increasing in developing countries, with varied prevalence between rural and urban areas [63]. Ethnic groups such as South Asians and Africans tend to develop T2D about a decade earlier and with a lower body mass index [64,65]. Screening services for T2D are not equally distributed between developing and developed countries. This highlights the urgent need for a simple, effective, and affordable screening method. In this study, we introduced the PHR as one of the blood-derived inflammatory indices linked to hyperglycemia and elevated HbA1c levels in diabetic patients. Unlike inflammatory cytokines like IL-6, IL-8, IL-1β, and TNF-α, the PHR ratio is easy to calculate from peripheral blood tests, which are affordable, convenient, and straightforward to interpret. Hence, PHR has the potential to be used as a universal, non-invasive, and cost-effective test to assess diabetic risk in obese patients.

The findings presented in this study showed a significant association between PHR and glycemic markers in diabetic obese patients and suggested that PHR can be used as a potential marker for dysglycemia among T2D patients to reduce its severity and mortality. Our study has a few limitations. This is a single-centered study, which restricts the generalizability of the results. Given the retrospective nature of this study, it was not possible to determine a causal relationship between elevated PHR values and disturbances in glucose metabolism in diabetes. Therefore, additional research is necessary to determine the role of the PHR in predicting T2D in obese patients.

## 5. Conclusions

In conclusion, PHR is elevated in obese patients when T2D is present. This observation is likely due to the significant association of PHR and dysglycemia. Our findings underscore the validity of the blood-derived inflammatory ratio such as PHR, an easily obtainable routine test, in predicting T2D in individuals with obesity. Further longitudinal studies are needed to elucidate the fundamental molecular mechanisms responsible for this association to gain insights into alternative prophylactic and therapeutic intervention strategies.

## Figures and Tables

**Table 1:** Baseline characteristics of the studied subjects.

Variables	All Subjects (n = 203)	Obese (n = 103)	Obese + T2D (n = 100)	*p*-Value
Demographics				
Age (years),	48 (35–58)	37 (29–46)	58 (51–65)	<0.0001
Sex (female), n (%)	141 (69.4%)	73 (70.8%)	68 (68.0%)	-
BMI (kg/m^2^),	35 (33–38)	36 (34–38)	35 (32–40)	0.4322
Lab Parameters				
RBC count (×10^12^/μL)	4.88 (4.49–5.31)	4.90(4.53–5.41)	4.80 (4.40–5.10)	0.0676
Hb (g/dL)	13.0 (12.00–14.70)	13.2 (11.98–14.73)	13.0 (12–14.25)	<0.0001
Hct (L/L)	27.70 (25.60–29.00)	27.40 (25.15–29.25)	28.0 (26.0–29.0)	0.2346
WBC (×10^9^/μL),	7.5 (6.0–7.0)	7.1 (5.5–8.3)	8.1 (6.6–9.4)	0.0002
Neutrophils (×10^3^/μL)	3.77 (2.76–5.00)	3.49 (2.29–4.61)	4.00 (3.07–5.22)	0.0065
Lymphocytes (×10^3^/μL)	2.75 (2.20–3.32)	2.62 (2.16–3.12)	3.00 (2.29–3.80)	0.0085
Monocytes (×10^3^/μL)	0.55 (0.44–0.68)	0.54 (0.40–0.65)	0.57 (0.50–0.74)	0.0080
Basophils (×10^3^/μL)	0.05 (0.03–0.07)	0.04 (0.03–0.06)	0.05 (0.04–0.07)	0.0035
Eosinophils (×10^3^/μL)	0.18 (0.11–0.28)	0.18 (0.11–0.25)	0.20 (0.11–0.32)	0.1343
ALT (U/L)	17 (13–26)	16 (12–25)	18 (14–26)	0.1152
PLT count (×10^6^/mL)	312.0 (265.3–369.8)	299.0 (255.5–363.3)	327.5 (272.3–378.3)	0.1166
FBG (mg/dL)	97.30 (90.1–140.1)	90.09 (82.9–93.7)	131.5 (97.3–162.2)	<0.0001
HbA1c (%)	6.2 (5.7–7.4)	5.7 (5.5–5.9)	7.4 (6.7–8.7)	<0.0001
TC (mg/dL)	177.9 (147.7–211.3)	196.8 (172.1–227.8)	154.7 (135.3–185.6)	<0.0001
TG (mg/dL)	115.1 (88.6–156.8)	103.6 (77.1–150.6)	124.0 (88.6–159.4)	0.0393
HDL (mg/dL)	43.7 (38.5–52.4)	47.6 (39.8–59.6)	40.8 (36.9–46.4)	<0.0001
LDL (mg/dL)	108.9 (85.1–136.8)	127.2 (106.1–152.1)	92.8 (69.6–116.0)	<0.0001
PHR	7.06 (5.71–8.785)	6.05 (4.75–7.72)	7.91 (6.39–9.44)	<0.0001
Comorbidities				
Hypertension, n (%)	60 (29.5%)	13 (12.6%)	47 (47%)	-
Hypothyroidism, n (%)	18 (8.8%)	16 (15.5%)	2 (2%)	-
DLD, n (%)	96 (47.2%)	19 (18.4%)	77 (77%)	-
Smoking, n (%)	22 (10.8%)	17 (16.5%)	5 (5%)	-
Medications				-
Metformin, n (%)	104 (51.2%)	11 (10.6%)	93 (93%)	-
GLP-1 agonist, n (%)	57 (28%)	31 (30%)	26 (26%)	-
SGL2 inhibitor, n (%)	34 (16.7%)	0 (0%)	34 (34%)	-

Abbreviations: BMI (body mass index); RBC (Red blood cells); Hb (hemoglobin); Hct (hematocrit test); WBC (white blood count); ALT (Alanine transaminase); PLT (Platelet); FBG (Fasting blood glucose); HbA1c (Glycated hemoglobin); TC (Total cholesterol); TG (Triglycerides); HDL (High-density lipoprotein); LDL (Low-density lipoprotein); PHR (Platelet/high-density lipoprotein cholesterol ratio); DLD (Dyslipidemia); GLP-1 (Glucagon-like peptide-1); SGL2 (Sodium–glucose cotransporter-2).

**Table 2:** Prevalence of normal and elevated PHR levels among the studied population.

Parameter	Non-Diabetic Obese	Diabetic Obese
N-PHR	67.07%	29.90%
H-PHR	32.93%	70.10%

**Table 3:** Risk assessment of elevated PHR and T2D.

	Score	95% CI	Z Statistic	Significance Level
RR				
Total subjects	2.13	1.52 to 2.97	4.42	*p* < 0.0001
Obesity class I	2.12	1.23 to 3.67	2.71	*p* = 0.0067
Obesity class II	1.6	0.86 to 2.97	1.49	*p* = 0.1345
Obesity class III	0.58	0.41 to 0.81	3.12	*p* = 0.0018
OR				
Total subjects	4.77	2.53 to 8.99	4.83	*p* < 0.0001
Obesity class I	5.81	2.01 to 16.78	3.25	*p* = 0.0011
Obesity class II	5.62	1.87 to 16.83	3.08	*p* = 0.002
Obesity class III	0.15	0.0074 to 3.16	1.21	*p* = 0.225

## Data Availability

Data are contained within the article and Supplementary Materials.

## Supplementary Materials

The following supporting information can be downloaded at: https://www.mdpi.com/article/10.3390/healthcare12151540/s1, Table S1: Baseline characteristics based on obesity classes.
