# Supplementary material for: Platelet/High-Density Lipoprotein Ratio (PHR) Predicts Type 2 Diabetes in Obese Patients: A Retrospective Study
Source: Healthcare (Basel). 2024 Aug 3;12(15):1540. doi: 10.3390/healthcare12151540 (PMC11311744; doi:10.3390/healthcare12151540)
Supplement: Supplementary file 1 [file healthcare-12-01540-s001.zip › healthcare-3070937-supplementary.pdf]

Supplemental Table S1. Baseline characteristics based on obesity classes

Variables	Class I	Class II	Class III	*P value*
**Obese subjects**				
Demographics				
Age (years)	38 (31-46)	35 (27-45)	35 (29.5-43.5)	*0.54*
Sex (female), n (%)	25 (34.25%)	40 (54.79%)	8 (10.96)	-
BMI (kg/m²)	33 (32-34)	37 (35-38)	41 (40-42.5)	*<0.0001*
Prevalence %	34.0	53.4	12.6	-
Lab Parameters				
RBC count (×10⁶/μL)	4.89 (4.57-5.38)	4.81 (4.48-5.42)	4.98 (4.71-5.53)	*0.527*
Hb (g/dL)	13.2 (12-15.2)	13.2 (11.93-14.73)	13.5 (11.6-14.65)	*0.929*
Hct, (L/L)	0.41 (0.377-0.45)	0.411 (0.38-0.44)	0.42 (0.38-0.45)	*0.878*
WBC (×10⁹/L)	7.4 (5.8-8.4)	6.4 (5.07-8.00)	6.80 (5.90-8.85)	*0.336*
Neutrophils (×10³/L)	3.66 (2.74-4.41)	3.24 (1.99-4.63)	3.24 (2.31-5.19)	*0.789*
Lymphocytes (×10³/μL)	2.71 (2.37-3.34)	2.51 (2.02-2.99)	2.86 (2.52-3.23)	*0.154*
Monocytes (×10³/μL)	0.55 (0.39-0.67)	0.52 (0.39-0.64)	0.58 (0.48-0.67)	*0.273*
Basophiles (×10³/μL)	0.04 (0.03-0.06)	0.04 (0.03-0.06)	0.05 (0.04-0.075)	*0.246*
Eosinophil (×10³/μL)	0.18 (0.11-0.23)	0.18 (0.085-0.25)	0.18 (0.13-0.33)	*0.721*
ALT(U/L)	17 (11-24)	15 (12.75-26)	19 (13.5-31.5)	*0.548*
PLT count (×10⁶/mL)	313 (252-365)	294 (255-346.8)	305 (275.5-433)	*0.44*
FBG (mg/dl)	91.89 (84.68-93.69)	88.29 (81.53-93.69)	91.89 (83.78-104.10)	*0.356*
HbA1c(%)	5.76 (5.39-6.14)	5.63 (5.47-5.85)	5.75 (5.39-5.86)	*0.504*
TC (mg/dl)	203.4 (171.4-231.8)	197.6 (170.5-227.8)	190.8 (173.6-223)	*0.878*
TG (mg/dl)	109.4 (92.33-156.3)	101 (73.51-125.8)	115.1 (81.93-168.5)	*0.471*
HDL (mg/dl)	44.86 (37.80-56.26)	48.34 (40.99-59.94)	46.02 (39.54-62.36)	*0.602*
LDL (mg/dl)	129.5 (112.3-162.2)	126.8 (106.3-151.2)	116.8 (91.94-149.6)	*0.398*
PHR	5.93 (4.64-8.24)	6.13 (4.72-7.54)	6.47 (5.59-7.81)	*0.813*
Medications				
Metforminn, n (%)	5 (45.5%)	5 (45.5%)	1 (9.09%)	-
GLP-1 agonist, n (%)	9 (29.03%)	18 (58.06%)	4 (12.9%)	-
SGL2 inhibitor, n (%)	0 (0%)	0 (0%)	0 (0%)	-
**Obese+T2D subjects**				
Demographics				
Age (years)	55.5 (51.25-65.75)	58.5 (53.5-64.5)	58 (45.75-61.75)	0.399
Sex (female), n (%)	29 (42.65%)	17 (25%)	22 (32.35%)	-
BMI (kg/m²)	32 (31-33)	37 (36-38)	43.5 (41-45.5)	<0.0001
Prevalence %	48	26	26	-
Lab Parameters				
RBC count (×10⁶/μL)	4.90 (4.40-5.40)	4.80 (4.45-5.00)	4.50 (4.32-4.90)	0.175
Hb (g/dL)	14 (12-15)	14 (12-14)	13 (11-13)	0.055
Hct, (L/L)	0.42 (0.38-0.47)	0.42 (0.39-0.45)	0.40 (0.38-0.425)	0.168
WBC (×10⁹/L)	7.80 (6.20-9.40)	8.45 (7.00-9.225)	8.90 (7.20-10.70)	0.089
Neutrophils (×10³/L)	3.60 (2.80-5.20)	4.25 (3.70-5.00)	4.40 (3.25-5.90)	0.1
Lymphocytes (×10³/μL)	2.90 (2.20-3.40)	2.85 (2.35-3.80)	3.30 (2.75-4.10)	0.17
Monocytes (×10³/μL)	0.55 (0.48-0.74)	0.60 (0.50-0.75)	0.60 (0.47-0.72)	0.813
Basophiles (×10³/μL)	0.05 (0.04-0.08)	0.06 (0.03-0.07)	0.05 (0.04-0.07)	0.962
Eosinophil (×10³/μL)	0.21 (0.10-0.30)	0.2 (0.11-0.32)	0.21 (0.11-0.30)	0.836
ALT(U/L)	20 (16-26)	19 (14.5-25)	16 (13.75-31.25)	0.358
PLT count (×10⁶/mL)	331 (264-384)	308 (257-371)	338 (301-388)	0.216

FBG (mg/dl)	134.2 (105-175.7)	113.5 (90.09-162.2)	124.3 (109-158.6)	0.539
HbA1c(%)	7.8 (7-9.5)	7.4 (6.67-8.72)	7.1 (6.5-8.25)	0.228
TC (mg/dl)	154.7 (131.5-177.9)	148.9 (134.4-202.1)	170 (149.1-189.5)	0.209
TG (mg/dl)	132.9 (88.57-177.1)	119.6 (88.57-141.7)	115.1 (88.57-146.1)	0.356
HDL (mg/dl)	38.67 (34.42-42.54)	43.89 (38.67-49.69)	46.4 (42.15-52.78)	<0.0001
LDL (mg/dl)	85.07 (65.74-112.1)	88.94 (72.51-128.6)	100.5 (83.14-116.00)	0.3238
PHR	8.34 (6.99-10.68)	7.20 (5.71-8.485)	7.29 (5.91-9.08)	0.031
Medications				
Metforminn, n (%)	45 (48.39%)	23 (24.73%)	25 (26.88%)	-
GLP-1 agonist, n (%)	12 (46.15%)	5 (19.23%)	9 (34.62%)	-
SGL2 inhibitor, n (%)	22 (64.71%)	6 (17.65%)	6 (17.65%)	-

Abbreviations: BMI (body mass index); RBC (Red blood cells); Hb (hemoglobin); Hct (hematocrit test); WBC (white blood count); ALT (Alanine transaminase); PLT (Platelet); FBG (Fasting blood glucose); HbA1c (Glycated hemoglobin); TC (Total cholesterol); TG (Triglycerides); HDL (High-density lipoprotein); LDL (Low-density lipoprotein); PHR (Platelet/high-density lipoprotein cholesterol ratio); DLD (Dyslipidemia); GLP-1(Glucagon-like peptide-1); SGL2 (Sodium-glucose cotransporter-2).
